# Food Safety Practices and Associated Factors among Food Handlers of Fiche Town, North Shewa Zone, Ethiopia

**DOI:** 10.1155/2021/6158769

**Published:** 2021-12-17

**Authors:** Samuel Chane Teferi, Israel Sebsibe, Birhanu Adibaru

**Affiliations:** Salale University, Department of Biology, P.O. Box 245, Fiche, Ethiopia

## Abstract

**Background:**

Foodborne diseases remain a major public health problem globally, but the problem is severe in developing countries like Ethiopia. The objective of this study was to assess food safety practices and associated factors among food handlers of Fiche town.

**Methods:**

A cross-sectional study was conducted among 422 food handlers working in food and drink establishments. The data were collected using a structured questionnaire and observational checklist. Data were entered and coded into SPSS for analysis. Multivariable logistic regression was used to identify the predictor variables associated with the practice of food handlers (*p* < 0.05).

**Result:**

61.6% of food handlers knew the potential risk of contaminating food with dirty hands, and 70% washed hands with soap before working with food. 52.8% of food handlers covered the hair with restraints. 66.8% of food handlers used outer garments, and the majority of food handlers (71.1%) had a trimmed fingernail. Two hundred thirteen (50.5%) of food handlers had good food handling practices. Medical checkup (AOR = 3.16; 95% CI 1.89, 5.26), sanitary inspection (AOR = 1.76; 95% CI 1.16, 2.69), knowledge (AOR = 2.31; 95% CI 1.53, 3.48), service year (AOR = 3.11; 95% CI 1.53, 6.31), and educational status (AOR = 3.42, 95% CI 1.29, 9.04) were found to be significantly associated with food handling practices.

**Conclusion:**

The food handlers should take various training concerning food hygiene and safety to enhance their knowledge and practice. Regular sanitary inspection of food and drink establishments is recommended.

## 1. Introduction

Foodborne diseases remain a major public health problem globally and are responsible for significant morbidity and mortality rates [1]. According to the World Health Organization (WHO), more than 600 million illnesses and 420,000 annual deaths worldwide are due to contaminated food, and about 33 million disability-adjusted life years are attributable to foodborne infections globally [2]. Foodborne diseases are severe in developing countries due to difficulties securing optimal hygienic food handling practices and poor sanitation [3, 4]. Lifestyle change and urbanization lead people to dine away from home more often, contributing to the unregulated opening of eating establishments with inadequate hygiene conditions [5]. Estimating the magnitude of foodborne disease is complicated because reliable statistics are unavailable in most countries due to poor or nonexistent reporting systems [6]. Approximately 10–20% of foodborne illnesses outbreaks are because of contamination by food handlers [7]. Like other developing countries, foodborne diseases are prevalent in Ethiopia; the country's annual incidence of foodborne illnesses ranged from 3.4 to 9.3%, the median being 5.8% [8].

Microbiological contamination of food occurs in catering establishments due to poor personal hygiene practices, inappropriate storage temperature, and dirty food contact surfaces [9]. Food handlers may be infected by a wide range of enteropathogens and engaged in transmitting many infections to the public. These intestinal parasites and enteropathogenic bacteria transmit directly or indirectly through objects contaminated with feces [10–12]. *Salmonella* and *Shigella* are among the common causes of foodborne diseases throughout the world [13]. Food handlers are anyone who directly handle packaged or unpackaged food, food equipment, and utensils (such as cutlery, plates, bowls, or chopping boards) [14].

There are many factors associated with food handling practices. The major contributing factors for potential foodborne pathogen outbreaks in food and drink establishments are due to inadequate knowledge of food handlers [15–17], poor educational status [18], insanitary conditions of food and drink establishments [17, 19], and marital status [16, 20]. Studies in different parts of Ethiopia showed poor food safety practices due to the factors mentioned above and weak regulatory systems, inadequate food safety laws, and lack of financial resources to invest in safer equipment in the country [15, 16, 21].

According to Kibret and Abera [17] and Kumie and Zeru [22], good personal hygiene and food handling practices are the basis for preventing the transmission of pathogens from food handlers to consumers. Therefore, to reduce foodborne illnesses, it is crucial to understand the knowledge and practices of food handlers [23]. The number of food and drink establishments in Fiche town increases from time to time, and many people use the food and drink services in the city. Moreover, food safety practices and their associated factors among food handlers were unknown in the town. Hence, the present study aimed to assess food safety practices and associated factors among food handlers working in food and drink establishments in Fiche town. Since the study was cross-sectional, it will not show a cause and effect relationship, and the study might be liable to social desirability and recall bias.

## 2. Methods

### 2.1. Description of the Study Area

**Figure 1:**
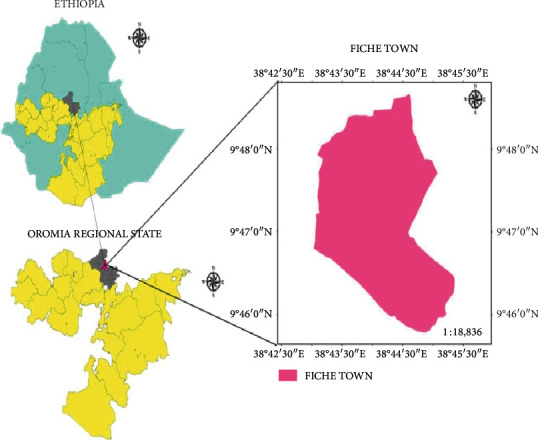
Map of the study area (source: ARC GIS database).

Fiche is a town in central Ethiopia. It is the North Shewa Zone of Oromia Region's administrative center and has four kebeles (Figure 1). It is located about 114 km north of Addis Ababa, the capital city of Ethiopia. Fiche has a latitude and longitude of 9°48′N and 38°44′E, respectively. Fiche town has an elevation between 2,738 m and 2,782 m above the sea level.

### 2.2. Study Design

A community-based cross-sectional study was conducted to evaluate food handlers' practices and associated factors working in food and drink establishments in Fiche town.

### 2.3. Population and Sampling Procedures

Food handlers working in food and drink establishments such as hotels, café and restaurants, snack houses, restaurants, juice houses, and butcher shops in the study area were the focus of the study. The number of food handlers was challenging to estimate, according to the tourism office data. A simple random sampling technique was used to select food and drink establishments. Then, actively working food handlers were selected randomly. The sample size was determined using a single population proportion formula. By taking the proportion (*p*) of 50%, since there is no previous investigation in the town, significance level 5% (*α* = 0.05), *Zα*/2 = 1.96, the margin of error between the population and the sample 5% (*d* = 0.05), and finally, 10% nonresponse rate was considered.

(1) $$\begin{array}{c} n={\left(\frac{Z\alpha }{2}\right)}^{2}P\frac{\left(1-P\right)}{d^{2}},\\ n={\left(1.96\right)}^{2}\times 0.5\frac{\left(1-0.5\right)}{{\left(0.05\right)}^{2}}=384. \end{array}$$

Therefore, the final sample size was 422.

### 2.4. Data Collection Tools and Procedures

The data collection instrument used for this study includes a structured questionnaire and observational checklist adopted from related literature in Ethiopia and elsewhere. The questionnaire was first prepared in English, translated to local languages, and translated back to English to see any inconsistency. The pretest was performed on 5% of food handlers outside the sampling town; then, correction and modification were undertaken based on the gaps identified during the pretest. Data collectors and supervisors were oriented about the purpose of the study, the components of the questionnaire, and data quality management. The questionnaire was used to assess food handlers' sociodemographic characteristics (age, sex, educational status, marital status, service year, and income) and knowledge (cause, mode, and symptom of foodborne diseases). Observational checklists were used to assess food handlers' practice while they performed their task on-site. Respondents were assessed using 12 practice-related checklists to evaluate the level of practice. Food handlers who scored less than or equal to the mean value (≤50%) were considered as having a “poor level of practices,” and those who scored greater than the mean value (>58.3%) were considered as having a “good level of practices” [16, 17, 21]. Respondents were asked 8 knowledge-based questions to assess the level of knowledge. Respondents who scored less than or equal to the mean value (≤50%) were considered as having a “poor level of knowledge,” and those who scored greater than the mean value (>62.5%) were considered as having a “good level of knowledge” [16, 17, 24].

### 2.5. Study Variables

Food safety practice was the dependent variable in this study, and educational level, age, gender, work experience, medical checkup, marital status, income, knowledge, and sanitary inspection were the independent variables.

### 2.6. Data Analysis

Consistency and completeness of data were checked and verified during collection, entry, and analysis. Statistical Package for Social Sciences (SPSS) version 26 was used to analyze the data. Bivariate logistic regression was used to identify variables with *p* value <0.25. Then, these variables were analyzed in multivariable logistic regression to assess the independent effect after controlling other variables [25]. The model fitness was checked by the Hosmer and Lemeshow goodness-of-fit test and was found to be 0.590. Finally, a *p* value less than 0.05 was considered for determining statistically significant variables.

### 2.7. Ethical Considerations

The study was undertaken after approval by Salale University's research and community service directorate. During data collection, verbal and written informed consent was also obtained from study participants after the purpose of the study was explained. We kept the confidentiality of the respondents and for the food and drinking establishments by asking the participants not to write their names on the questionnaires and codes to hide the identity of the food and drinking establishments.

## 3. Result

### 3.1. Sociodemographic Characteristics of Food Handlers

The majority, 302 (71.6%), of food handlers were females, and 120 (28.4%) were males. 5.7% of respondents were illiterate. Most of the food handlers (63.7%) were single, while 32.2% of them were married, and 199 (47.2%) had an income of 1001–1500 Ethiopian birr (46 ETB = 1 USD). Only 42 (9.9%) food handlers had over ten years of service years (Table 1).

### 3.2. Knowledge of Food Handlers

The majority, 354(83.9%), of food handlers had heard about foodborne diseases. Mass media (33.9%) was the most common source of information, followed by friends and customers (28.2%). The proportion of food handlers who believed that foodborne diseases are caused by contamination with bacteria and parasites was 280 (66.4%), and 208 (49.3%) responded that contaminated water was the channel for transmissions. Of those asked about the mode of transmission of foodborne disease, 248 (58.8%) answered that contaminated food was the vehicle. 260 (61.6%) of food handlers knew the potential risk of contaminating food with dirty hands (Table 2).

### 3.3. Practice of Food Handlers on Food Hygiene

Among the total food handlers observed during visits, 282 (66.8%) used outer garments. The majority, 210 (74.5%), of food handler's outer garments were clean. 223 (52.8%) of food handlers covered hair with restraints. The majority of food handlers, 300 (71.1%), had a trimmed fingernail.

### 3.4. Factors Associated with Food Handling Practice

Two hundred thirteen (50.5%) food handlers had good food handling practices. Multivariable logistic regression analysis revealed that medical checkup (AOR = 3.16; 95% CI 1.89, 5.26), sanitary inspection (AOR = 1.76; 95% CI 1.16, 2.69), knowledge (AOR = 2.31; 95% CI 1.53, 3.48), service year (AOR = 3.11; 95% CI 1.53, 6.31), and educational status (AOR = 3.42; 95% CI 1.29, 9.04) were found to be significantly associated with food handling practices with *p* value <0.05 (Table 3).

## 4. Discussion

Foodborne diseases remain a major public health problem across the globe. Still, the problem is severe in developing countries like Ethiopia due to difficulties securing optimal hygienic food handling practices [3]. Therefore, to reduce foodborne illnesses, it is crucial to understand the knowledge and practices of food handlers [23]. In this study, 213 (50.5%) had good food safety practices from 422 food handlers, and 49.5% had poor food safety practices. Our finding is lower than studies conducted in Indonesia (90%) [26], Bahir Dar (67.6%) [27], Jordan (89.4%) [28], Mekelle (63.9%) [6], and Malaysia (59.3%) [24]. The variation might be due to study settings and food safety culture. According to Nyarugwe et al. [29], taking food safety culture into account is a promising way to improve food safety performance in the food industry. However, the present finding was comparable with studies conducted in Debark town (40.1%) [30], Dangila town (52.5%) [16], Gondar town (49%) [15], and Nigeria (50%) [31]. The possible reason for discrepancies might be the difference in the study design, cutoff points, food handler's sociodemographic profile, and year of study.

Food handlers with a good level of knowledge were 2.31 times more likely to have good food safety practices than those with a poor level of knowledge (AOR = 2.31; 95% CI 1.53, 3.48). Our finding is supported by studies conducted in Gondar, Malaysia, Dangila, Mekelle, and Gondar town [1, 6, 15, 16, 32]. Having adequate knowledge regarding foodborne disease transmission mechanisms and ways of food contamination is essential to undertake particular basic practices to alleviate those problems [33]. But in the present study, only 149 (35.3%) of food handlers had good knowledge of food safety. The present finding is higher than a study conducted in Dangila town (28.8%) [16] but lower than a study conducted in Gondar (44.3%) [15], India (58.3%) [34], and Malaysia (51.6%) [35]. This might be due to differences in the study setting, development of researched site, and sociodemographic profile of respondents. Food safety practice was significantly associated with sanitary inspection. The probability of having good food safety practice was 1.76 times higher among food handlers who were supervised than their counterparts (AOR = 1.76; 95% CI 1.16, 2.69). The present finding was supported by a study conducted in Arba Minch and Gondar [15, 21]. This might be due to supervisors giving advice and feedback to food handlers, the owners, and the managers during an inspection.

Food handlers who had medical checkups were 3.16 times more likely to have good food handling practices than those who had no medical checkups (AOR = 3.16; 95% CI 1.89, 5.26). This could be the healthcare workers who advised food handlers during the examination, enhancing their food handling practice. The present finding is supported by studies conducted in Arba Minch, Dessie, and Gondar town [15, 21, 36]. In the present study, service year (experience) was significantly associated with food safety practice. The odds of performing good food handling practice among food handlers who had >10 years' experience was 3.11 times higher than those who had less than one year experience (AOR = 3.11; 95% CI 1.53, 6.31). In line with our finding, a study from Bahir Dar, Debark, and Gondar town reported that experienced staff had good food handling practice (AOR: 3.4; 95% CI 1.8, 6.4; AOR: 1.95; 95% CI 1.11, 3.45; AOR: 3.3703; 95% CI 1.2943, 8.7846) [1, 27, 30]. The possible explanation might be food handlers acquired better knowledge and skills through repeated exposure. Similarly, the odds of performing good food handling practices among food handlers who have attained above grade twelve education were 3.42 times more likely to have good food handling practices than illiterates (AOR = 3.42; 95% CI 1.29, 9.04). The possible explanation might be the depth of knowledge could affect food handlers' food handling practices. The present finding is comparable with previous studies [18, 27, 28, 30]. In the present study, food hygiene training was not significantly associated with food handling practice. In contrast to our study, other reports showed a significant association between training and food handling practices [1, 15, 21, 27]. The possible reason might be that most food handlers did not take food hygiene training (80.3%) in this study. In agreement with our finding, a similar result was reported from Mekelle, Bahir Dar, and Dangila town [5, 16, 17].

Of the 282 respondents who used outer garments in the present study, 210 (74.5%) of the food handler's outer garments were clean. The present finding is comparable with a study conducted in Mekelle town (72.6%) [22]. A higher result was reported in a study conducted in Bahir Dar (92.6%) [17] and Dangila town (88.7%) [16]. 223 (52.8%) food handlers covered their hair with restraints in our study. The present study is comparable with a study conducted in Gondar town (51.5%) [15]; however, it is much better than similar studies conducted in Zeway (40.1%) [37] and Mekelle (39%) [22].

Previous studies on handwashing compliance ranged 5%–50% among food handlers in food service facilities [38]. In line with previous investigations, 295 (70%) food handlers wash hands with soap before working with food in our finding. A higher result was reported in South Africa, who found that 94% washed their hands under all circumstances [39]. Compared to other hand parts, the area beneath fingernails harbors most microorganisms and is the most difficult to clean [40]. Therefore, to prevent the transmission of foodborne diseases, food handlers working in food and drink establishments should trim their fingernails. In the present study, most food handlers 300 (71.1%) had a trimmed fingernail. Our finding is supported by a study conducted in Ghana (63.3%), Aligarh (69.9%), and Mekelle town (76.2%) [22, 41, 42].

## 5. Conclusion and Recommendations

The present study revealed that there was poor food handling practice among food handlers. The predominant factors associated with good food handling practices were medical checkups, educational status, sanitary inspection, knowledge, and service year. The food handlers should take various training concerning food hygiene and safety to enhance their knowledge and practice. Moreover, a regular sanitary inspection of food and drink establishments is recommended. Future studies should focus on the enumeration of bacteria from food utensils and food handlers.

## Figures and Tables

**Table 1:** Sociodemographic characteristics of food handlers working in food and drink establishment in Fiche town (*n* = 422).

Characteristics	Frequency	Percentage (%)
Sex		
Male	120	28.4
Female	302	71.6

Age		
≤20	92	21.8
21–30	274	64.9
31–40	49	11.6
>40	7	1.7

Educational status		
Illiterate	24	5.7
Grades 1–6	125	29.6
Grades 7–12	179	42.4
Grades >12	94	22.3

Marital status		
Single	269	63.7
Married	136	32.2
Divorced	17	4

Service year of food handlers		
<1 year	161	38.2
1–5 years	174	41.2
6–10 years	45	10.7
>10 years	42	9.9

Food handler's monthly income (in birr)		
500–1000	97	23
1001–1500	199	47.2
>1500	126	29.8

**Table 2:** Knowledge status of food handlers on food handling practices (*n* = 422).

Variable	Frequency	Percentage (%)
Heard about foodborne disease		
No	68	16.1
Yes	354	83.9

If yes, your source of information (*n* = 354)		
Health center	64	18.1
Sanitarian during inspection	70	19.8
Mass media	120	33.9
Others (friends and customers)	100	28.2

Cause of foodborne disease^*∗*^		
Contaminated with bacteria and parasites	280	66.4
Adding chemicals	90	21.3
Anger of god	2	0.5
Unhygienic food preparation	250	59.2

Mode of transmission of foodborne disease^*∗*^		
Contaminated food	248	58.8
Contaminated water	208	49.3
Vectors (flies and cockroaches)	50	11.8

Reason for food contamination^*∗*^		
Contact of unhygienic hands	260	61.6
Unhygienic working environment	211	50
Unclean utensils	199	47.2
Infected food handlers	48	11.4
Exposure to insects and rats	6	1.4

Symptoms of foodborne disease^*∗*^		
Vomiting	189	44.8
Fever	140	33.2
Diarrhea	277	65.6
I do not know	33	7.8

Germs found on cutting board		
Yes	295	69.9
No	127	30.1

Good personal hygiene prevents foodborne disease		
Yes	378	89.6
No	32	7.6
I do not know	12	2.8

^*∗*^Because of the possibility of multiple responses, the total number of food handlers may not be equal to 422 (100%).

**Table 3:** Determinants of food safety practice among food handlers working in food and drinking establishments in Fiche town.

Variables	Food safety practice		Wald	Sig.	AOR (95% CI)
	Good	Poor			
Sanitary inspection					
Yes	161	133	7.05	0.008^*∗*^	1.76 (1.16, 2.69)
No	52	76	1		

Medical checkup					
Yes	64	25	19.54	0.001^*∗*^	3.16 (1.89, 5.26)
No	149	184	1		

Knowledge					
Good	95	54	15.95	0.001^*∗*^	2.31 (1.53, 3.48)
Poor	118	155	1		

Service year					
<1 year	59	102	1		
1–5 years	88	86	5.57	0.018^*∗*^	0.27 (0.09, 0.80)
6–10 years	39	6	2.5	0.113	1.75 (0.87, 3.5)
>10 years	27	15	9.87	0.002^*∗*^	3.11 (1.53, 6.31)

Educational status					
Illiterate	7	17	1		
1–6	53	72	0.35	0.552	1.16 (0.70, 1.93)
7–12	98	81	5.52	0.019^*∗*^	1.91 (1.11, 3.29)
>12	55	39	6.17	0.013^*∗*^	3.42 (1.29, 9.04)

^*∗*^Statistically significant at *p* < 0.05.

## Data Availability

The datasets used to support the findings of this study are available from the corresponding author upon request.

## Abbreviations

AOR: Adjusted odds ratio
ETB: Ethiopian birr
CI: Confidence interval
WHO: World Health Organization
SPSS: Statistical Package for Social Sciences.
